# NTMS based tractography and segmental diffusion analysis in patients with brainstem gliomas: Risk stratification and clinical potential

**DOI:** 10.1016/j.bas.2024.102753

**Published:** 2024-02-09

**Authors:** Lion Weiß, Fabia Roth, Pierre Rea-Ludmann, Tizian Rosenstock, Thomas Picht, Peter Vajkoczy, Anna Zdunczyk

**Affiliations:** a Charité – Universitätsmedizin Berlin, Department of Neurosurgery, Germany; b Cluster of Excellence Matters of Activity. Image Space Material, Humboldt Universität zu Berlin, Germany; c Berlin Institute of Health at Charité – Universitätsmedizin Berlin, Germany

**Keywords:** Brainstem gliomas, Tractography, Transcranial magnetic stimulation, Diffusion tensor imaging, Fractional anisotropy, Fiber tracking

## Abstract

**Introduction:**

Surgery on the brainstem level is associated with a high-risk of postoperative morbidity. Recently, we have introduced the combination of navigated transcranial magnetic stimulation (nTMS) and diffusion tensor imaging (DTI) tractography to define functionally relevant motor fibers tracts on the brainstem level to support operative planning and risk stratification in brainstem cavernomas.

**Research question:**

Evaluate this method and assess it's clinical impact for the surgery of brainstem gliomas.

**Material and methods:**

Patients with brainstem gliomas were examined preoperatively with motor nTMS and DTI tractography. A fractional anisotropy (FA) value of 75% of the individual FA threshold (FAT) was used to track descending corticospinal (CST) and -bulbar tracts (CBT). The distance between the tumor and the somatotopic tracts (hand, leg, face) was measured and diffusion parameters were correlated to the patients’ outcome.

**Results:**

12 patients were enrolled in this study, of which 6 underwent surgical resection, 5 received a stereotactic biopsy and 1 patient received conservative treatment. In all patients nTMS mapping and somatotopic tractography were performed successfully. Low FA values correlated with clinical symptoms revealing tract alteration by the tumor (p = 0.049). A tumor-tract distance (TTD) above 2 mm was the critical limit to achieve a safe complete tumor resection.

**Discussion and conclusion:**

nTMS based DTI tractography combined with local diffusion analysis is a valuable tool for preoperative visualization and functional assessment of relevant motor fiber tracts, improving planning of safe entry corridors and perioperative risk stratification in brainstem gliomas tumors. This technique allows for customized treatment strategy to maximize patients’ safety.

## Highlights

• Navigated transcranial magnetic stimulation (nTMS) based diffusion tensor tractography was combined to identify functionally relevant motor pathways non-invasively in brainstem glioma patients.
• A detailed diffusion analysis was used to investigate tract integrity and structural impairment of the brainstem.
• Low risk-factors e.g., the threshold for gross total resections at a tumor-tract distance of >2 mm were discovered, eventually leading to a remission of preoperative symptoms.
• High-risk factors like lower peritumoral fractional anisotropy values or infiltrative tumors attributed to worsened postoperative outcome.
• Our techniques enhanced the planning of safe entry corridors and perioperative risk stratification in brainstem tumors and allowed for individual patient counselling and customized treatment strategy to maximize patients' safety.

## Abbreviations

ADC: Apparent diffusion coefficient
CBT: Corticobulbar tract
CN: Cranial nerve
CST: Corticospinal tract
DTI: Diffusion tensor imaging
DTT: Diffusion tensor tractography
FA: Fractional anisotropy
FAT: FA threshold
FACT: Fiber assignment by continuous tracking
HGBSG: High-grade brainstem gliomas
LGBSG: Low-grade brainstem gliomas
MEP: Muscle evoked potentials
RMT: Resting Motor Threshold
ROI: Regions of interests
SEP: Sensory evoked potentials
TTD: Tumor-tract distance

## 1 Introduction

Brainstem tumor surgery is controversial and very heterogenous in terms of postoperative outcome. Perioperative morbidity and worsened postoperative neurological outcome account for 10–30% of patients (Babu et al., 2014) (Mursch et al., 2005) (Teo and Siu, 2008) (Kesari et al., 2008). The mean survival rates depend significantly on the histopathology and vary between 11.2 months for high-grade brainstem gliomas (HGBSG) and 7.3 years for low-grade brainstem gliomas (LGBSG) (Guillamo et al., 2001) (Reyes-Botero et al., 2012) (Babu et al., 2014).

Additional therapy modalities like radiotherapy do not have a great influence on HGBSG in terms of survival rates (Dey et al., 2014) (Guillamo et al., 2001). Conversely, in LGBSG radiation can lead to stable disease and improvement of symptoms in 62% vs. 13% for HGBSG (Dey et al., 2014) (Guillamo et al., 2001). However also here a variety of complications is associated with the treatment (Kesari et al., 2008).

To provide patients with the best counsel for surgery, it is crucial to identify and stratify preoperative factors that influence the postoperative outcome.

Recently, we have shown the value of nTMS based diffusion tensor tractography (DTT) in brain tumor surgery (Frey et al., 2014). Equally important are improved surgical techniques (intraoperative navigation, monitoring) that enable a precise localization of the tumor, vulnerable structures and to define save entry zones (Constantini and Epstein, 1996) (Yang et al., 2019) (Cavalcanti et al., 2016). Especially in adults with HGBSG, radiation therapy is often not sufficient, therefore surgery had a positive impact on the time of survival (9 vs. 6 months) (Dey et al., 2014). Moreover tumor resection could lead to a nearly doubled time of survival compared to biopsy alone (42 vs. 22 months, Babu et al., 2014).

NTMS based DTI tractography has already proven to be beneficial in preoperative planning in brainstem cavernoma surgery, in order to define safe entry zones and stratify the risk for neurological deterioration. However, this technique has not yet been investigated in patients with brainstem gliomas (Zdunczyk et al., 2019) (Flores et al., 2015) (Januszewski et al., 2016).

In this study we therefore aim to identify the clinical potential of preoperative nTMS based DTT in brainstem tumor surgery to improve surgical strategies and postoperative outcome. To accomplish this goal the individual tractography results, segmental diffusion MRI parameters and clinical outcomes were analyzed.

## 2 Methods

### 2.1 Patients

All patients gave their informed consent to take part in this study. The study was designed to fulfill the ethical principles according to the latest Helsinki Declaration from July 9, 2018.

The following patients’ characteristics were acquired through examinations and treatments that occurred between April 2014 to December 2019 (including follow-ups): age, sex, motor status, pre-/postoperative symptoms, surgical intervention, histology, tumor level, tumor size, tumor site, surgical approach, 12-months survival time.

**Fig. 1:**
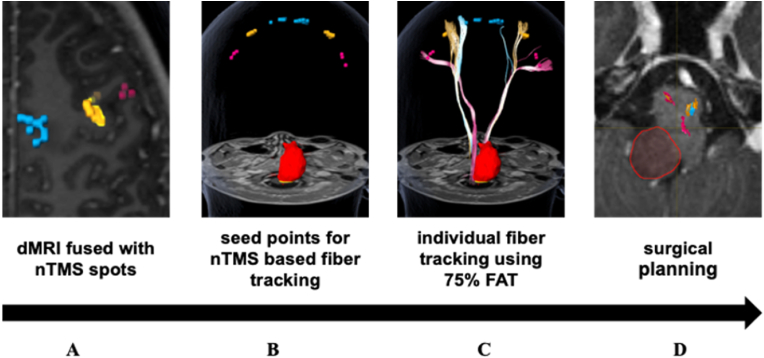
Preoperative protocol. The preoperative protocol starts with the MRI scan (including diffusion weighted imaging and DTI), followed by the nTMS examination for determination of primary motor area (**A&B**) and seed points, nTMS based fiber tracking (**C&D**) and the surgical planning.

All patients underwent the same preoperative protocol (Fig. 1, Zdunczyk et al., 2019).

### 2.2 MRI protocol

For the initial diagnostics a cranial MRI scan was carried out on a 1.5 T or a 3 T MRI scanner (GE Healthcare, Milwaukee, Wis, Siemens 3 T). The section thickness was 1.0-mm. T1 inversion recovery (TR/TE/TI/α 7.8 ms/3.1 ms/500 ms/16°), 3D gradient-echo sequence (IR 3D-FSPGR). A diffusion weighted single-shot echo-planar sequence along 23 different geometric directions at a b-value of 1000 s/mm^2^ was repeated to sample a diffusion tensor. The scan parameters were TR/TE 11,000 ms/83 ms; matrix size 128 × 128; and FOV 240 × 240 mm (Zdunczyk et al., 2019).

### 2.3 NTMS examination

The nTMS sequences followed a specific standardized protocol as described before (Fig. 1, Krieg et al., 2012).

This technique allows us to detect the functionally relevant motor areas on the precentral gyrus. The EMG responses for the hand, leg and facial motor areas were recorded while a biphasic magnetic stimulus was applied. The spots on the cortical motor area with true-positive EMG responses were imported as seed points to the fiber tracking software. The same number of positive nTMS seed points were selected for each extremity. As a measure of cortical excitability the Resting Motor Threshold (RMT) was calculated by an algorithm using the FDI muscle as the target muscle (Zdunczyk et al., 2019).

Before every nTMS examination a neurological examination was performed including a finger tapping test and hand dynamometer testing. Standardized questionnaires like the EORTC QLQ-C30 (Version 3) and the Karnofsky Performance Scale were used to assess the patients’ everyday abilities. The individual motor strength was examined by a neurosurgeon preoperatively and postoperatively using the Medical Research Council Scale (0–5) after 7 and 30 days.

### 2.4 Tractography

An individual diffusion tensor tractography of the descending motor fibers was created for each patient with “Elements” fiber tracking software (2.0, Brainlab, München) using a modified fiber assignment by continuous tracking (FACT) algorithm (Oishi et al., 2010). Here a standardized protocol was used, developed by members of the Image Guidance Lab, Berlin (Fig. 1). (Frey et al., 2012) (Rosenstock et al., 2017).

The following parameters were collected for every patient through this process: average FA values, FAT, tumor-tract distances (CST/CBT), min./max. fiber length, average fiber length, fiber angulation.

**Fig. 2:**
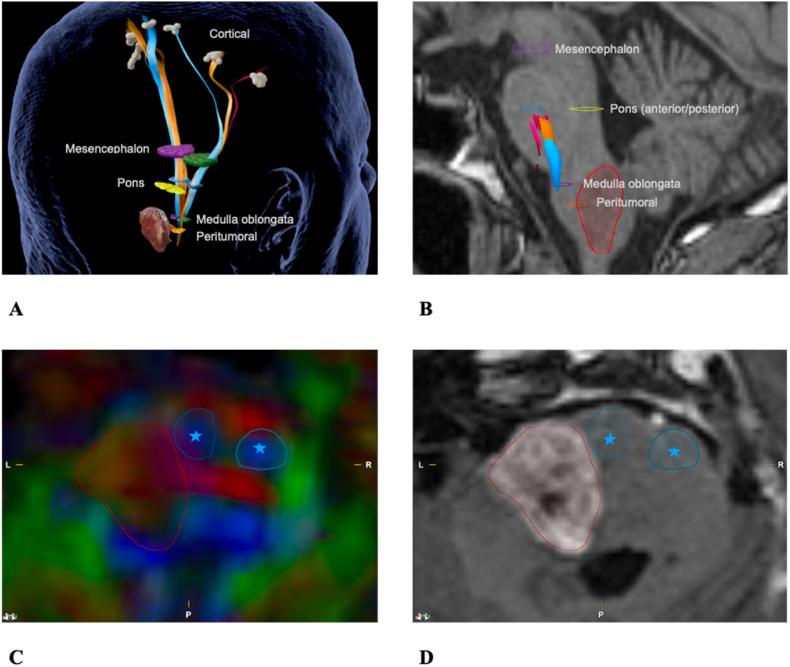
ROI setting. **A**: Overview of all ROIs, the descending motor fiber tracts and the tumor (patient #7). Color code: Blue for leg (CST), Orange for hand (CST) and Pink for face (CBT) fibers. **B**: Sagittal T1 (patient #7): overview of all ROIs in the right hemisphere; red boundaries mark the tumor. **C-D**: Patient #12 example of segmental ROI setting. The ROIs (blue stars) are in the anterior Pons and the borders of the tumor (marked in red); MRI sequences: 2C – FA map, 2 D – T1-weighted MP-RAGE

For visualization of the tracts a consistent color code was used (Fig. 2A, 7–9).

#### 2.4.1 Specifics of tractography

The structural and diffusion MRI sequences (T1 MP-RAGE, DTI, ADC, B0) were imported to the fiber tracking software. All sequences and measured ‘positive’ nTMS points on the motor cortex were fused with the T1 MP-RAGE sequence. Resulted image distortions from the fusion process were corrected with the software.

Regions of interests (ROIs) were used, to selectively visualize the fibers correlating with the CST and CBT. Therefor the ‘positive’ nTMS points and the Crura cerebri functioned as ROIs (Conti et al., 2014). The placement of the ROIs was based on anatomical landmarks in the T1-weighted MRI and the color-coded FA map (Fig. 2, Weiss et al., 2015; Rosenstock et al., 2017).

The FA value was increased until no fibers could be depicted. This indicated the FAT threshold, of which 75% were the basis for the fiber tracking (Fig. 1). (Frey et al., 2012) The fiber length was set to a minimum of 110 mm with a maximum angulation of 30°.

The created fiber tracts were characterized according to their course (normal, deviated, partially interrupted, interrupted) and the spatial relation to the resection cavity was noted (Table 1). (Li et al., 2018) (Lazar et al., 2006) The boundaries and sizes of the tumors as well as the distances between the tumor and tracts could be measured using the planning software. Eventually the individual tractography was used by the neurosurgeons to create a customized preoperative plan and surgical strategy.

#### 2.4.2 Analysis of diffusion imaging

The colored-FA sequences and the ADC sequences were analyzed to measure the diffusion in different anatomical regions. Using iPlan (3.0; Brainlab, München) the FA value and ADC value were calculated in the different segmental ROIs which were set bilaterally in the Medulla oblongata, Pons (anterior and posterior), Mesencephalon and peritumoral region depending on the location of the tumor (Fig. 2A–B). For precise orientation a split view of the colored FA and T1 MP-RAGE was used to set the segmental ROIs (Fig. 2C–D). The segmental ROIs were the same size within each brainstem level.

Together the FA or ADC values of the Mesencephalon, anterior Pons and Medulla oblongata represented the mean ‘Brainstem anterior’ FA or ADC values, respectively. The positive nTMS spots on the motor cortex represented the cortical ROIs.

The data was divided in ‘affected’ and ‘contralateral’ groups. ‘Affected’ hereby refers to the tract or ROI located on the ipsilateral hemisphere of the tumor whereas ‘contralateral’ refers to the contralateral hemisphere.

### 2.5 Surgical intervention

During the surgery the fiber tracking was displayed in the operative theater to the neurosurgeons supported by neuronavigation (Picht et al., 2011) (Kombos et al., 2009). Intraoperative navigation was also enhanced by electrophysiological neuromonitoring, e.g. muscle evoked potentials (MEPs), sensory evoked potentials (SEPs) and free running EMG of the cranial nerves (CN) (Kombos et al., 2009) (Sala, 2017).

### 2.6 Data analysis and gathering

The statistical analysis was performed using SPSS Statistics (27.0.0.0; IBM, Armonk). The visualization of the graphs was accomplished using Prism 9 (9.1.0; GraphPad Software, San Diego).

All presented p-values derive from 2-sided t-testing. ‘Cohen's d’ was calculated for significant t-testing to estimate the effect sizes and will be further presented as ‘d’ (Cohen, 1992). For the analysis of more than 2 grouped variables, ANOVA was used. The Pearson correlation was applied to calculate the relation between groups.

Inclusion criteria was a symptomatic brainstem tumor that was treated within our neurosurgical department. Exclusion criteria were incomplete MRIs, incomplete DTI studies and missing nTMS data.

## 3 Results

### 3.1 Characteristics

**Table 1:**
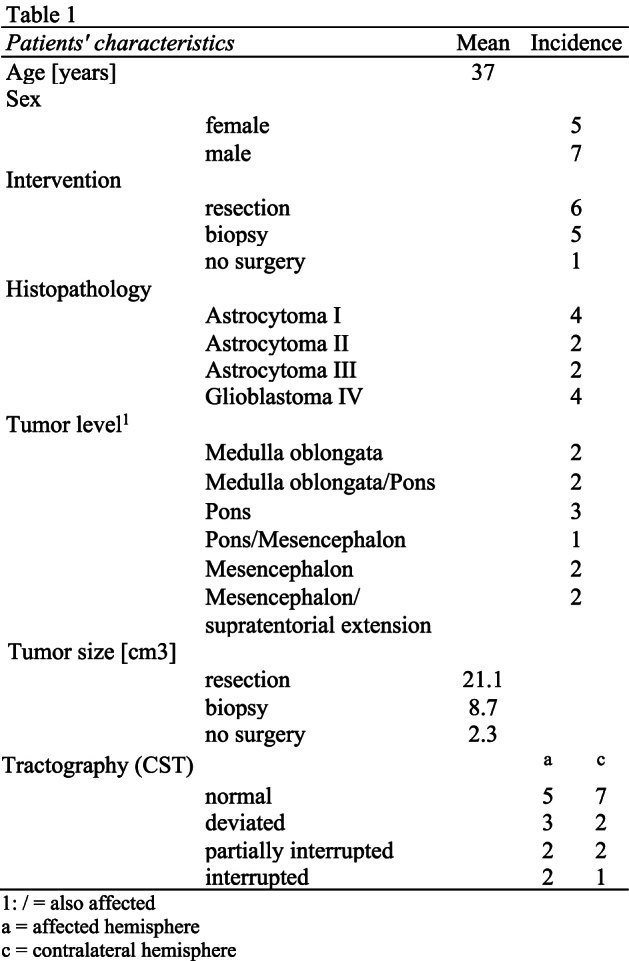
Shows the characteristics of the 12 patients, aged 15–60 years (mean 37yrs., 5 females), that were included to this study.

### 3.2 Symptoms

**Fig. 3:**
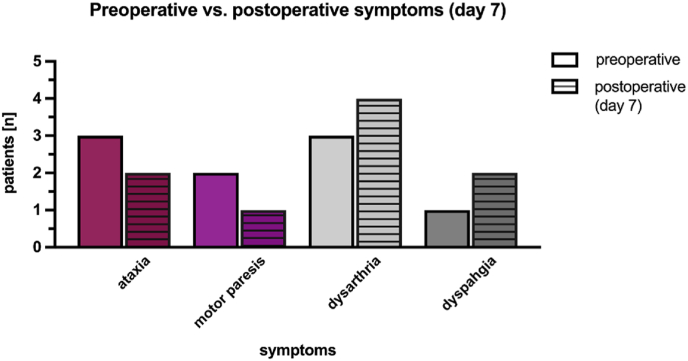
Overview of preoperative vs. postoperative symptoms. The figure includes a selection of the most frequent symptoms.

Fig. 3 shows a selected overview of the main pre- and postoperative symptoms. The symptoms most frequently found were cranial nerve deficits followed by ataxia and paresthesia of the extremities.

The total number of symptoms after surgical resection decreased by 18,5%. No patient developed a new motor deficit of the extremities.

The main new postoperative symptoms were dysarthria and dysphagia, vegetative dysfunction and impaired consciousness (Fig. 3).

### 3.3 Surgical outcome

**Fig. 4:**
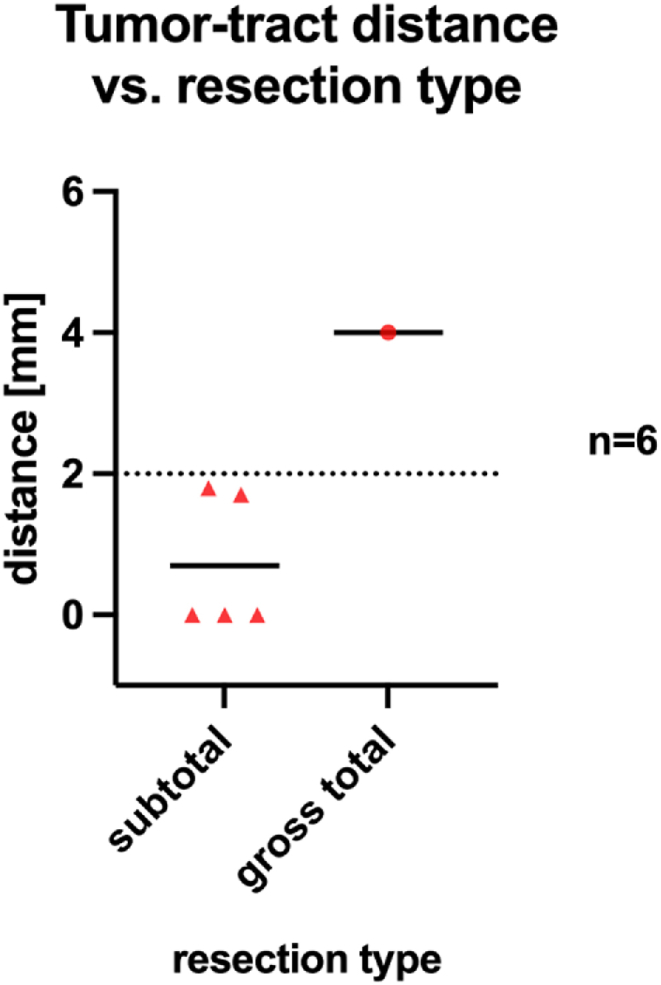
Comparison of tumor-tract distance to resection type.

Of all resected tumors, 1 patient had a gross total resection. In the remaining only a subtotal resection could be achieved due to abnormalities in the intraoperative monitoring. Here, a decrease of >30 % in MEP amplitude (compared to baseline), increase in stimulation intensity of 30 mA or a repeated C train in the free running EMG of the cranial nerves was treated as a warning sign during surgery. In 33% of patients a significant reduction of MEPs (>50% amplitude) occurred. In these patients the preoperatively defined tumor-tract distance (TTD) was below 2 mm (Fig. 4).

One patient in the resection group died after 3 months and one patient in the biopsy group died after 5 months. Both patients had a diagnosed Glioblastoma IV. All other patients lived longer than the follow up period of 12 months.

### 3.4 nTMS based tractography & diffusion

#### 3.4.1 RMT & FAT

**Fig. 5:**
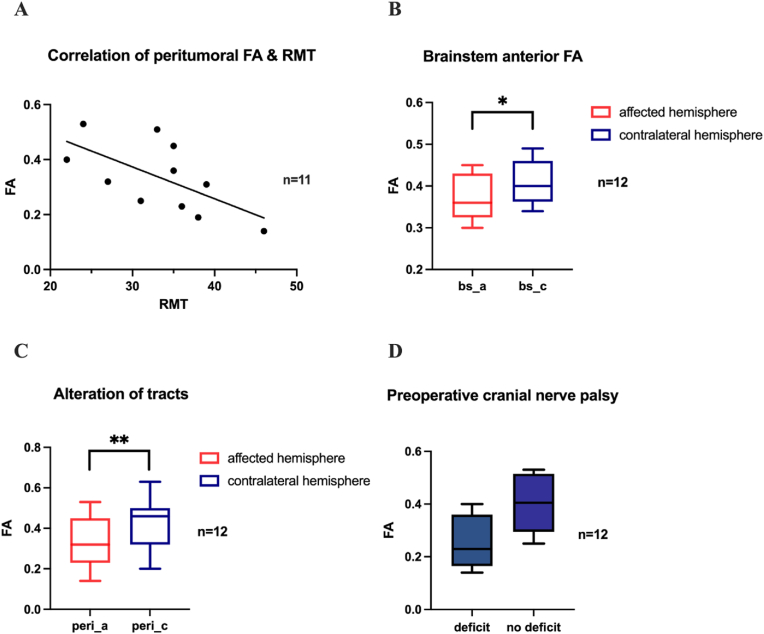
Diffusion analysis. **A**: Negative correlation of peritumoral FA values and RMT (p < 0.05; r = −0.630, n = 11). **B:** Comparison of FA values between the hemispheres in the anterior brainstem (p = 0.01; d = 0.41, n = 12). **C:** Comparison of peritumoral FA values between the hemispheres (p < 0.01; d = 0.88, n = 12). **D**: Comparison of peritumoral FA values between patients with preoperative cranial nerve palsy and without this deficit (p = 0.053, n = 12).

On average the mean RMT was lower on the affected than on the contralateral hemisphere. A significant negative correlation was found between the RMT and the peritumoral FA on the affected hemisphere of all patients (Fig. 5A).

#### 3.4.2 Segmental diffusion analysis

There was a significant difference of FA values between the affected and contralateral hemisphere in the anterior brainstem (Fig. 5B).

The peritumoral FA values (p = 0.008, d = 0.88) and cortical FA values (p = 0.048, d = 0.64) reached a significant decrease in the affected hemisphere (Fig. 5C).

In this patient group also the peritumoral ADCs (p = 0.036, d = 0.73; data not shown) and the ADC values of the anterior brainstem including the peritumoral values (p = 0.042, d = 0.67; data not shown) revealed a significant difference.

In patients with cranial nerve symptoms the preoperative peritumoral FA was lower than in patients without these symptoms (0.25 vs. 0.40, p = 0.052).

#### 3.4.3 Fiber tract analysis

In all patients a nTMS mapping and a somatotopic DTI tractography was performed successfully.

The number of deviated to interrupted fiber tracts was higher on the affected (n = 8) than on the contralateral hemisphere (n = 6) (Table 1). In patients with deviated fiber tracts the RMT was higher (mean = 35.3; SD = 7.06) when compared to patients with normal tractographies (mean = 30.2; SD = 7.14; p = 0.221).

The different courses of the contralateral CST were also compared to the mean distance between CST and the tumor. A significant decrease in distance was found from normal to interrupted tracts (ANOVA: p = 0.033). Here, these deformed and interrupted fiber tracts revealed a close spatial relationship to the tumor border with a tumor tract distance of 0 mm.

In total six patients showed a TTD of 0 mm to the CST, of which three experienced preoperative motor disturbances i.e., paresis or ataxia. When analyzing the sensitivity and specificity of TTD with 0 mm in patients with preoperative motor disturbances, the results showed a sensitivity of 100% and a specificity of 59% (data not shown).

### 3.5 Case examples

**Fig. 6:**
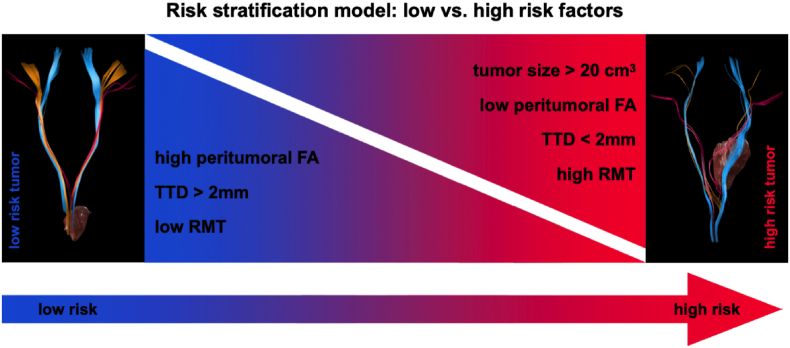
Risk stratification model of low vs. high-risk factors. Low risk factors include high peritumoral FA values, tumor-tract distances (TTD) >2 mm and low RMT values. High risk factors include tumor sizes >20 cm^3^, low peritumoral FA values, TTD <2 mm and high/increased RMT values.

The following cases exemplify how segmental diffusion and alteration of nTMS based fiber tracts go along with the clinical symptoms and risks (Fig. 6).

**Fig. 7:**
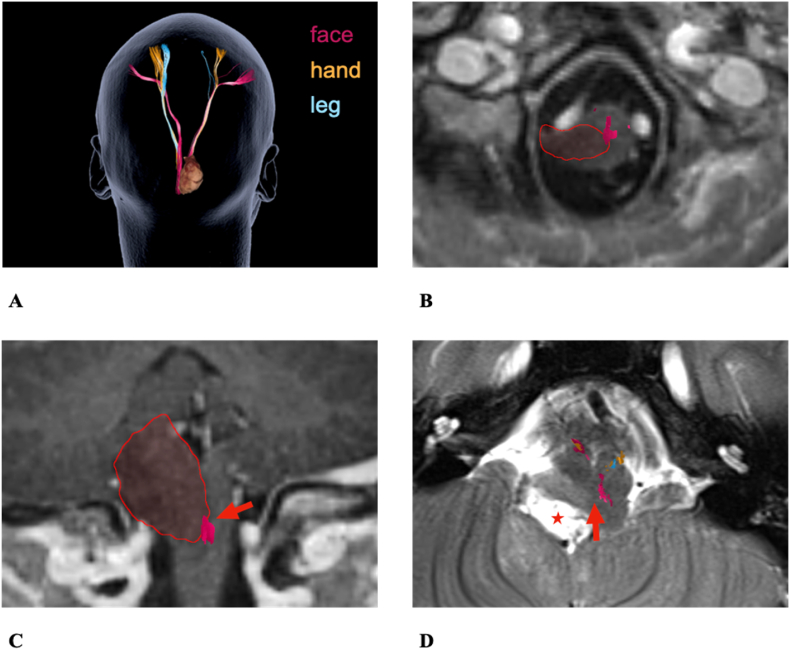
Case example patient #9. **A**: Overview of the descending motor fibers and tumor. **B**: Axial preoperative T1: The tumor (marked in red) in the posterior right Medulla oblongata infiltrating the CBT. **C**: Coronary preoperative T1: The tumor (marked in red) in the posterior right Medulla oblongata infiltrating the CBT (red arrow). **D**: Axial postoperative T2: The resection cavity (red star). The red arrow indicates the close distance to the CBT. Subtotal resection due to affection of the caudal cranial nerves in the intraoperative neuromonitoring

1) 15 years old female patient #9 with a Pilocytic Astrocytoma located in the right Medulla oblongata (Fig. 7)

Preoperative symptoms: dysphagia and hoarseness.

Postoperative symptoms: right sided hypoesthesia, disturbed fine motor skills, ataxia and dysphagia.

The distance between tumor and CBT was 0 mm in the Medulla oblongata (Fig. 7B). This fits the preoperative disturbances of lower cranial nerves with dysarthria and dysphagia and indicates the risk for aggravated dysphagia postoperatively.

There was a mismatch between FAs (affected hemisphere: 0.18 vs. contralateral: 0.32) and peritumoral ROIs (affected: 0.19 vs. contralateral: 0.38). Moreover, the disturbed tract integrity was found in the posterior Pons (affected: 0.31 vs. contralateral: 0.40). The RMT values were 38.00 (affected) and 40.00 (contralateral). These findings indicate a high-risk tumor (Fig. 6) and explain the postoperative transiently disturbed motor skills and the hypoesthesia.

However, with a CST distance of 9.00 mm the patient recovered well from all postoperative symptoms within a period of twelve months (Fig. 7C–D).

**Fig. 8:**
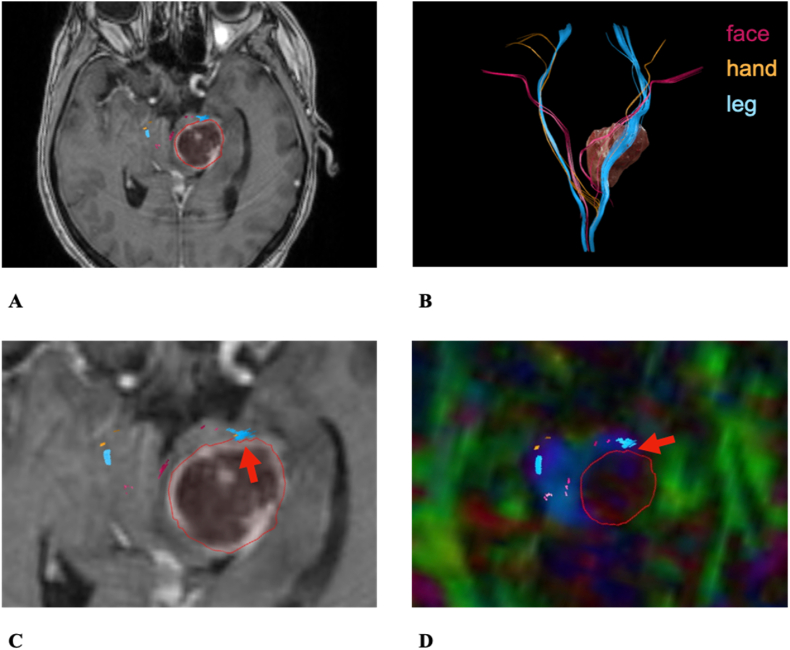
Case example patient #14. **A**: Axial preoperative T1 displaying the tumor (marked in red). **B**: Overview of the descending motor fibers and tumor. Partially interrupted fibers and strong deviation of the left CST. **C**: Axial preoperative T1: 0.00 mm tumor-tract distance to the left CST (red arrow). **D**: Axial preoperative FA-map: Disturbance of the left descending motor fibers (red arrow)

2) 59 years old female patient #14 with a Glioblastoma IV partially located in the left Mesencephalon (Fig. 8)

Preoperative symptom: right sided hemiparesis (MRC 2/5).

The partially interrupted and deviated left CST (0.00 mm TTD) shows an involvement of the descending motor fibers (Fig. 8B–C). Additionally, the segmental ROIs revealed a high mismatch between the affected and contralateral hemisphere when comparing the pontine level (affected: 0.43 vs. contralateral: 0.50) to the Mesencephalon (affected: 0.24 vs. contralateral: 0.47; decrease of 49%). Further, the RMT value was increased (affected: 35.00 vs. contralateral: 30.00). This constellation indicates a high-risk tumor (Fig. 6), so the surgical team opted for biopsy and the patient was treated conservatively.

**Fig. 9:**
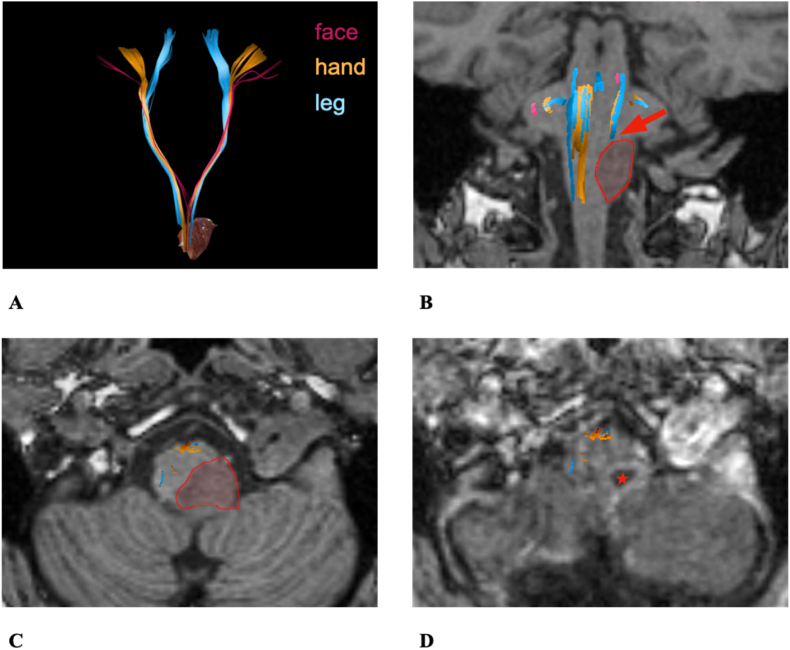
Case example patient #7. **A**: Overview of the descending motor fibers and tumor. **B**: Coronal preoperative T1: Interruption of the left pathways in the posterior Medulla oblongata (red arrow). **C**: Axial preoperative T1: The tumor (marked in red) in the posterior left Medulla oblongata and the descending motor fiber in the anterior Medulla oblongata. **D**: Axial postoperative T1: The resection cavity (red star) in the posterior left Medulla oblongata and the descending motor fiber in the anterior Medulla oblongata

3) 53 years old male patient #7 with an Astrocytoma II located in the left Medulla oblongata (Fig. 9)

Preoperative symptoms: diplopia, hoarseness, vestibular disorder.

Postoperative symptoms after surgery: ataxia, left sided hypoesthesia with left sided hyperalgesia and diplopia.

In the DTI visualization an interruption of descending tracts by the lesion was found (Fig. 9B). In parallel we also encountered low FA values (0.26) when compared to the contralateral side (0.31) as well as a high difference in peritumoral ROIs (affected: 0.23 vs. contralateral: 0.32) with a TTD of 4 mm. The RMT values were 36.00 (affected) vs. 34.00 (contralateral). This constellation indicates an alteration of descending tracts but with the TTD classifies as intermediate risk tumor and the patient was planned for surgery (Fig. 6).

## 4 Discussion

Even with novel operative techniques i.e., intraoperative navigation and neuromonitoring, surgical procedures on the brainstem level still carry a high risk for perioperative morbidity and mortality (Babu et al., 2014) (Mursch et al., 2005) (Teo and Siu, 2008). The identification and protection of vulnerable structures are a key element of preoperative planning and intraoperative determination of safe entry zones. As our results show this can be achieved by combining nTMS and DTI imaging preoperatively to gain valuable insights on the functional anatomy of the brainstem and therefore improve safety in brainstem tumor surgery.

In our study, infiltrative tumors presented with higher rates of postoperative symptoms. In 83.3% only a subtotal resection could be achieved due to close vicinity to functionally relevant pathways proved by an intraoperative decline (>50% compared to baseline) or loss of MEPs. These results are emphasized in Fig. 4, Fig. 7D.

### 4.1 Diffusion MRI in patients with brainstem tumors

In the last two decades the diffusion MRI modalities, like ADC and DTI, enabled a non-invasive stratification of impaired white matter pathways in pathologic neuroanatomy (Basser et al., 1994) (Le Bihan, 2003) (Salamon et al., 2005). Various studies have found that a disturbance in diffusion correlates with an alteration of supratentorial tracts and its coherent symptoms (Rosenstock et al., 2017) (Sener, 2001) (Zolal et al., 2013).

Lower FA values indicate a disturbance of the anisotropic diffusion that is found along white matter tracts, which is often used as a measure of fiber integrity (Sundgren et al., 2004).

Moreover, FA and ADC analysis have been used to evaluate the effect of radiation therapy and chemotherapy in pediatric patients with brainstem glioma (Prabhu et al., 2011) (Chen et al., 2010). Respectively, a coherent increase and decrease of FA and ADC values indicated a positive response to the therapy, which was also associated with a significant increase in survival (Prabhu et al., 2011) (Chen et al., 2010).

However, there are only few studies on preoperative DTI/ADC tract alteration in patients with infratentorial brain lesions, especially in adults (Helton et al., 2006) (Helton et al., 2008) (Flores et al., 2015).

#### 4.1.1 Segmental diffusion MRI analysis

One focus of this study was, to analyze diffusion MRI sequences (DTI, ADC) of the brainstem using different segmental ROIs of the nTMS based tractography and analyze their interhemispheric difference to further rule out tract integrity.

As described in Fig. 5B–C the affected hemisphere had lower FA values than the contralateral hemisphere on the level of the anterior brainstem and peritumoral area. Further the tract integrity was only disturbed in the brainstem, especially peritumoral, and not in the entire tracts. This emphasizes the structural impairment caused by the tumor. Moreover, it confirms the previous findings in supratentorial tumors where significant interhemispheric differences in diffusivity parameter, particularly adjacent to lesions have been described (Rosenstock et al., 2017).

#### 4.1.2 Diffusion based symptom mapping

In our study patients with a decrease of FA values of the CST or Lemniscal system showed coherent symptoms i.e., paresis or paresthesia, respectively (e.g., Fig. 7, Fig. 8, Fig. 9). To take another example, patients with preoperative cranial nerve disturbances presented with lower FA values in the peritumoral ROIs of the CBT (Fig. 5D). Since these patients are most frequently affected by cranial nerve symptoms, the FA analysis of the CBT is a valuable tool to quantify perioperative morbidity.

Considering our findings, a decrease in FA values might serve as an additional indicator of high risk of tract disturbance, eventually resulting in symptoms coherent to the associated region.

### 4.2 Tract integrity and cortical excitability

The results of the diffusion analysis were supported by the electrophysiological results of the nTMS examination. The peritumoral FA value had an influence on the cortical excitability as it correlated negatively with the RMT (Fig. 5A). In other words, the higher intensity needed to evoke a muscle potential, the lower was the tract integrity in the brainstem. These findings emphasize that a reduced fiber integrity is reflected by a lower cortical excitability. Our group has shown a similar negative correlation of the RMT and FAT in patients with brainstem cavernoma (Zdunczyk et al., 2019).

### 4.3 nTMS based tractography in patients with brainstem tumors

Numerous studies have pointed out the improvement of neurosurgical preoperative planning by DTT (Lazar et al., 2006) (Frey et al., 2014) (Januszewski et al., 2016) (Kovanlikaya et al., 2009) (Flores et al., 2015). Subsequently it has been shown that nTMS based tractography brings advantages compared to standard DTT alone (e.g. in terms of user variability and definition of functionally relevant fiber tracts) and improves the outcome of patients with brain tumors (Frey et al., 2014) (Krieg et al., 2012) (Conti et al., 2014) (Forster et al., 2015) (Raffa et al., 2019). Therefore, we aimed to take advantage of nTMS based DTI tractography in our study.

Still there are only few publications on DTT in patients with brainstem tumors, especially in adults (Kovanlikaya et al., 2009).

We accomplished our goal to create a reliable tractography of the descending motor fibers (CST, CBT) in every case, helping the neurosurgeons in preoperative planning and intraoperative navigation.

Further the tractography results were correlated with pre- and postoperative symptoms using TTD and deviation analysis. The TTD analysis revealed a high sensitivity for patients with preoperative motor disturbances. Here, the measured TTD to the corresponding CST was 0 mm in all four patients (100%). However, 0 mm TTD were also found in three other patients with gliomas without these symptoms indicating a lower specificity (57%). These findings go along with Kovanlikaya et al.‘s study (Kovanlikaya et al., 2009).

The fiber tract analysis emphasized, that the number of deviated to interrupted fiber tracts increases significantly, the closer the TTD gets. This tract alteration could be quantified by the average FA and RMT. The higher the tract disturbance or interruption, the greater the decrease of FA values or increase of the RMT was observed, respectively. Therefore, the deviation and interruption of fibers are concomitant with the findings of the diffusion analysis and vice versa. This underlines the quality of the tractographies and was most importantly in line with the risk for new or persistent postoperative deficits.

### 4.4 Risk stratification model

We managed to identify specific criteria that might have a crucial effect on the postoperative outcome in this patient group. Fig. 6 shows a schematic overview of the stratification criteria, which are divided into low and high-risk factors.

Some factors could be directly attributed to worsened postoperative outcome, like new postoperative deficits or lower time of survival. This accounts for patients with lower peritumoral FA values or strongly infiltrative tumors, especially Glioblastoma IV. Hence, these factors were categorized as high-risk factors.

Conversely, some factors were associated with a more beneficial outcome. We discovered a threshold for gross total resections at a TTD of >2 mm, eventually leading to remission of preoperative symptoms. Therefore, these criteria were labeled as low risk factors. Parameters such as low aFA and high RMT values correlated with disturbed tract integrity and coherent symptoms but could not be correlated significantly in this small patient group. However, we classified these factors as non-beneficial for postoperative outcome.

To create a more reliable and holistic risk stratification, all these factors should be evaluated together and adapted for each patient individually.

### 4.5 Limitations & perspective

Firstly, the relatively small sample size (n = 12) that was analyzed retrospectively. The basic limitations of DTT have been already described in detail and should always kept in mind, when this technique is applied (Sundgren et al., 2004). Further, the nTMS based fiber tracts only have a high sensitivity for motor pathways. However, patients in our study often suffered from sensory deficits, therefore it might be useful to create tractographies of the sensory pathways (e.g., medial lemniscus), as shown by Ulrich et al. (2014)

In our study only preoperative DTI studies were performed and analyzed. Postoperative DTI could be helpful to evaluate recovery of white matter tracts by comparing preoperative and postoperative FA values. Chen et al. could show a significant correlation between these parameters and survival time in oncological treatments (Chen et al., 2010). Moreover, this could give more insight on the impact of the different surgical approaches on white matter.

Lastly, this study provided many valuable results of DTT and diffusion analysis in brainstem tumor patients. Particularly the meticulous comparisons of tractography and diffusion parameters with preoperative and postoperative symptoms for every patient individually, but also for patient groups, were one of the major strengths of this study. Eventually this demonstrated that the technologies and methods analyzed, can help neurosurgeons to assess structural impairment preoperatively. We are convinced, this will lead to a better customized therapy for each patient.

## 5 Conclusion

NTMS based DTT is a valuable non-invasive tool to visualize functionally relevant motor pathways in patients with brainstem tumors. We were able to show that a disturbed tract integrity contributes to worse outcomes in patients with brainstem gliomas. The structural impairment was reflected by the nTMS based DTT results and could therefore help neurosurgeons in individual patient counselling, preoperative planning and risk stratification. Local diffusion analysis combined with nTMS based DTT has a great potential to contribute to a patient tailored treatment strategy and lower the risk of brainstem tumor surgery.

## Previous presentations

Online presentation (power point) of the abstract at DGNC 2021 (Congress of the German Society of Neurosurgery) on June 8, 2021.

## Author contributions

Conceptualization, design and supervision of the trial were operated by A. Zdunczyk, T. Picht, L. Weiβ and P. Vajkoczy. The data was obtained by L. Weiβ and F. Roth. The methodical procedures of nTMS and diffusion analysis were improved by P. Rea-Ludmann and T. Rosenstock. Analyzation and interpretation of the data were performed by L. Weiβ and A. Zdunczyk. The first draft of the manuscript was written by L. Weiβ. Figures and tables were created by L. Weiβ and A. Zdunczyk. All authors were involved in interpretation of the data and critical revision of the manuscript and approved the final report.

## Declaration of competing interest

The authors declare that the research was conducted in the absence of any commercial or financial relationships that could be construed as a potential conflict of interest. T. Picht has served as a speaker for Nexstim Oy but is not a contracted consultant.
